# Cost Effectiveness of Daclatasvir/Asunaprevir Versus Peginterferon/Ribavirin and Protease Inhibitors for the Treatment of Hepatitis c Genotype 1b Naïve Patients in Chile

**DOI:** 10.1371/journal.pone.0141660

**Published:** 2015-11-06

**Authors:** Constanza L. Vargas, Manuel A. Espinoza, Andrés Giglio, Alejandro Soza

**Affiliations:** 1 Centre of Clinical Research, Health Technology Assessment Unit, Pontificia Universidad Catolica de Chile, Santiago, Chile; 2 Department of Public Health, Pontificia Universidad Catolica de Chile, Santiago, Chile; 3 Hospital Sotero del Río, Puente Alto, Santiago, Chile; 4 Department of Gastroenterology, Pontificia Universidad Catolica de Chile, Santiago Chile; Università degli Studi di Palermo, ITALY

## Abstract

**Introduction:**

Daclatasvir and Asunaprevir (DCV/ASV) have recently been approved for the treatment of chronic hepatitis C virus infection. In association, they are more effective and safer than previous available treatments, but more expensive. It is unclear if paying for the additional costs is an efficient strategy considering limited resources.

**Methods:**

A Markov model was built to estimate the expected costs in Chilean pesos (CL$) and converted to US dollars (US$) and benefits in quality adjusted life years (QALYs) in a hypothetic cohort of naive patients receiving DCV/ASV compared to protease inhibitors (PIs) and Peginterferon plus Ribavirin (PR). Efficacy was obtained from a mixed-treatment comparison study and costs were estimated from local sources. Utilities were obtained applying the EQ-5D survey to local patients and then valued with the Chilean tariff. A time horizon of 46 years and a discount rate of 3% for costs and outcomes was considered. The ICERs were estimated for a range of DCV/ASV prices. Deterministic and probabilistic sensitivity analyses were performed.

**Results:**

PIs were extendedly dominated by DCV/ASV. The ICER of DCV/ASV compared to PR was US$ 16,635/QALY at a total treatment price of US$ 77,419; US$11,581 /QALY at a price of US$ 58,065; US$ 6,375/QALY at a price of US$ 38,710; and US$ 1,364 /QALY at a price of US$ 19,355. The probability of cost-effectiveness at a price of US$ 38,710 was 91.6% while there is a 21.43% probability that DCV/ASV dominates PR if the total treatment price was US$ 19,355. Although the results are sensitive to certain parameters, the ICER did not increase above the suggested threshold of 1 GDP per capita.

**Conclusions:**

DCV/ASV can be considered cost-effective at any price of the range studied. These results provide decision makers useful information about the value of incorporating these drugs into the public Chilean healthcare system.

## Introduction

Chronic Hepatitis C Virus (HCV) infection is a major cause of liver disease leading to important negative health consequences, mainly liver cirrhosis and hepatocellular carcinoma (HCC) [1]. There is also a global concern due to its high impact on the population´s health in terms of mortality, morbidity and opportunity costs [2]. According to the World Health Organization there are approximately 130 to 150 million people infected with the virus worldwide representing a global prevalence of 3% [3]. However, this estimate varies widely across countries depending on the geographical region. Whilst the prevalence in Latin America was estimated in 1.6%, it reaches 3.8% and 3.7% in central and East Asia respectively [4].

Unlike other chronic viral infections, hepatitis C is considered a curable disease if a state known as sustained virological response (SVR) is attained with adequate pharmacological treatment. For years, the only available treatment was Peginterferon alpha associated with Ribavirin (PR). This combination provided a SVR close to 40% in genotype 1 patients and it reported significant rates of serious adverse effects [5, 6]. Later, two first generation protease inhibitors (PIs) became available in 2011, Boceprevir and Telaprevir, which in association with PR reported better health outcomes but no improvement regarding adverse events [7, 8]. In addition, there is a group of patients who are ineligible or intolerants to these drugs, who do not have further treatment options. More recently, new drugs have been introduced in the market, which have reported SVR rates higher than 90% and low rates of serious adverse effects [9]. Most have been already incorporated as recommended alternatives in relevant treatment guidelines [10, 11].

Among these drugs, the association of daclatasvir plus asunaprevir (DCV/ASV) has demonstrated high efficacy measured as SVR rates in chronic hepatitis C genotype 1b patients. DCV is a first class direct acting antiviral that inhibits the non-structural protein NS5A complex [12]. On the other hand, ASV is a selective NS3 protease inhibitor with antiviral activity against genotypes 1, 4, 5 and 6 [13]. The multicohort study (HALLMARK-DUAL) assessed the efficacy of DCV/ASV in treatment-naïve, previously non-responders and ineligible/intolerant to interferon based regimens. SVR was achieved in 90% of treatment-naïve, 82% of non-responders and 82% of the ineligible/intolerant patients [14]. Furthermore, Kumada et al. [15] assessed the efficacy of DCV/ASV in Japanese patients that were ineligible/intolerant or non-responders to interferon based regimens. SVR was achieved in 87.4% of ineligible/intolerant and in 80.5% of non-responders with similar SVR rates when considering cirrhotic (90.9%) and non-cirrhotic patients (84%).

Since 2010, Hepatitis C became one of the 80 diseases of the Chilean health benefit plan, for which a package of services, including diagnostic, treatment and follow-up are legally guaranteed. Either private or public payers are forced to cover these services and technologies. Currently, in the case of Hepatitis C, the combination PR is the only therapy for which the public sector provides financial coverage.

As mentioned above, DCV/ASV has demonstrated to improve the SVR rate impacting on the course of the disease by reducing the number of complications, disease specific related deaths, and the incidence rate in the long run. In terms of costs, although there is still uncertainty regarding the future price of the drugs, it is expected to be substantially higher than the currently financed PR. The present study aims at assessing the cost-effectiveness of DCV/ASV versus PR (standard of care) and first generation PIs in order to inform about the relative value of these drugs for the Chilean health care system.

## Materials and Methods

### Study design

A cohort multi-state Markov model was built in Microsoft Excel® to determine the cost effectiveness of DCV/ASV versus PR and triple therapy with PIs from the perspective of the Chilean public healthcare sector. The analysis was restricted to untreated (naïve) patients diagnosed with HCV genotype 1b. The model was divided into two parts: first, it considered a decision tree that represents the treatment phase and; a second part, the Markov model (MONARCH “MOdelling the NAtural histoRy and Cost-effectiveness of Hepatitis”) to represent the natural history of hepatitis C and its complications [16]. The treatment phase was modelled using per week cycles and the natural history of the disease used annual cycles.

During the treatment phase, a hypothetical cohort initially entered the model allocated to any of the five fibrosis (F0-F4) states according to the METAVIR classification. The distribution was based on Chilean epidemiological data on HCV [17]. The treatment phase for each antiviral drug therapy, considered information regarding adverse events, treatment discontinuations, treatment costs, monitoring costs and health related quality of life. Based on these parameters and the efficacy estimates of each antiviral, patients could achieve SVR, fail to respond or discontinue treatment. At the end of the treatment phase, if patients discontinued or had detectable HCV-RNA (considered treatment failures), were returned to their original health state. If they achieved SVR, they transited to five possible SVR states depending on the original fibrosis state of the patient (SVR-F0-F4).

**Fig 1:**
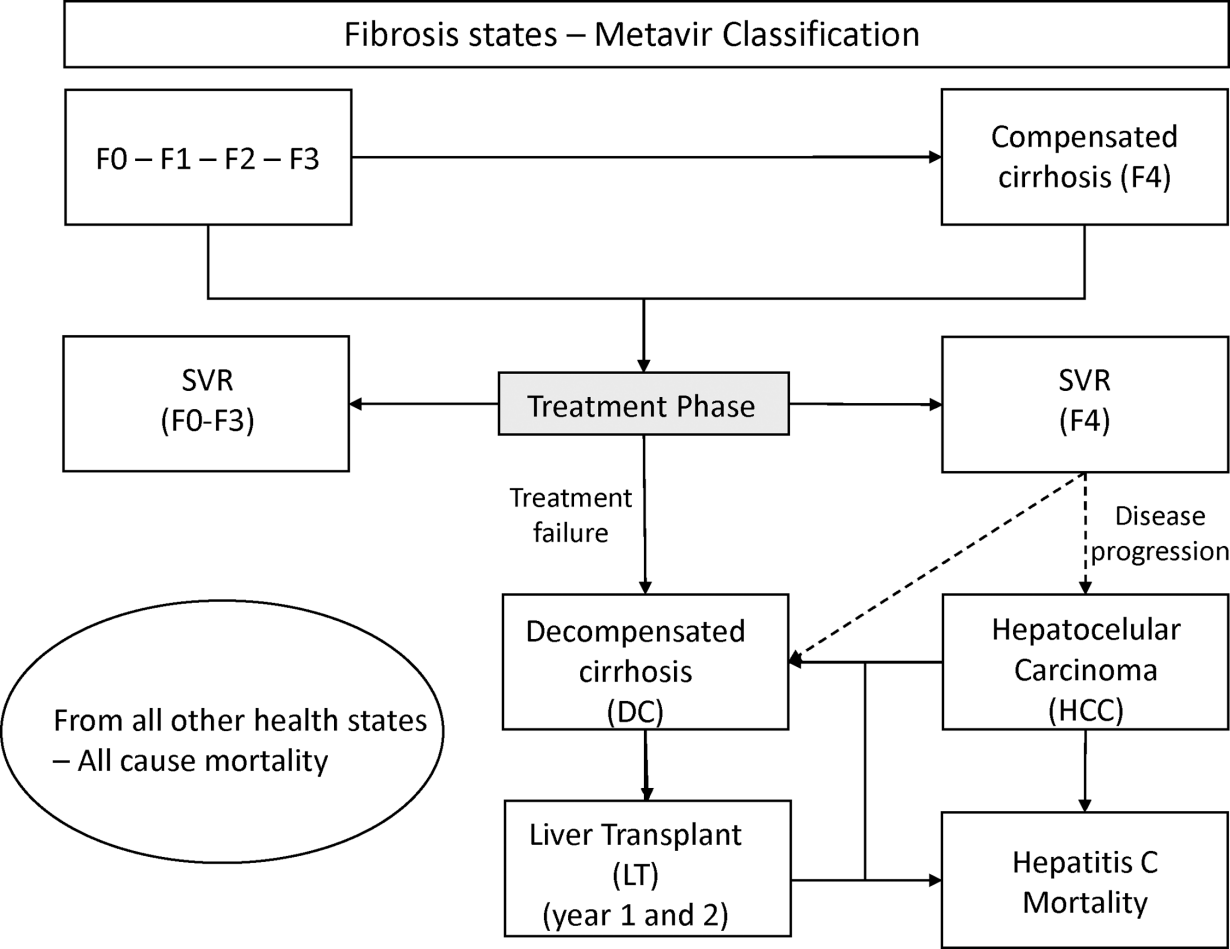
Markov State-transition decision model for Hepatitis C and liver disease: The model consists of two phases: treatment and natural history. If patients discontinue treatment due to adverse events or fail to respond and hence not achieve SVR, they enter the natural history component of the model, consisting of 15 health states: Fibrosis states (F0–F4); decompensated cirrhosis first (DC1) and subsequent years (DC2); hepatocellular carcinoma first (HCC1) and subsequent years (HCC2); liver transplant first (LT1) and subsequent years (LT2); disease specific mortality; mortality from all other causes (not shown); and SVR status states stratified by fibrosis stage (“SVR F0-F2”, “SVR-F3”, SVR-F4). For clarity, only liver transplant is stratified by state year. This figure is similar but not identical to the original image, and is therefore for illustrative purposes only.

After the treatment phase and depending on the initial health state of the patient and the treatment outcome, patients could transit to eleven possible health states that represent the natural history of the disease (Fig 1). Patients in health states representing complications of the disease, i.e. decompensated cirrhosis (DC) and hepatocellular carcinoma (HCC), could transit to liver transplant (LT). Furthermore, in order to account for the higher risk of mortality from these three states (DC, HCC and LT), a transition probability of hepatic related death was introduced into the model. The remaining patients were assumed to have the same risk of death as the general population, which was modelled using the Chilean population mortality rates [18].

### Model Assumptions

The Markov model represents a progressive disease where patients can either progress to more advanced stages or stay in that health state, but it does not allow patients to transit from more severe to less severe states. If patients in states F0-F3 transited to SVR, they were assumed cured. The model also assumed that these patients (SVR F0-F3) do not become symptomatic again. However, the model allows patients to transit from SVR-F4 to DC and HCC, since the eradication of the virus does not avoid the progression of a damaged liver. In order to account for mortality differences during the first versus subsequent years, one tunnel health state was introduced for states DC, HCC and LT.

The model also assumed that patients did not suffer disease progression while they were receiving the treatment, which is consistent with evidence that one patient may take 20 to 30 years until he/she develops cirrhosis since infection. In addition, it was also assumed that patients who received liver transplant (LT1-LT2) did not suffer disease progression; they only presented a higher risk of death.

### Input parameters

All model input parameters, their point estimates and standard error (SE) as well as the distribution assumed for the probabilistic sensitivity analysis are shown in Table 1. For parameters where the SE is unknown, the uncertainty was characterized assuming a 30% relative variation around the mean.

**Table 1:** Model Inputs for Hepatitis C Cost-Effectiveness Model: Base case, Standard Error and parameter distribution.

Variable		Mean	SE	Distribution	Reference
**Transition Probabilities**					
F0-F1		0.117	0.007	Beta	[21]
F1-F2		0.085	0.005	Beta	[21]
F2-F3		0.120	0.006	Beta	[21]
F3-F4		0.116	0.006	Beta	[21]
F4-DC		0.070	0.009	Beta	[22]
F4-HCC		0.022	0.011	Beta	[23]
DC-HCC		0.068	0.019	Beta	[24]
DC-LT		0.023	0.007	Beta	[25]
DC-DTHLR [year 1]		0.182	0.060	Beta	[22]
DC-DTHLR [year 2]		0.182	0.060	Beta	[22]
HCC-LT		0.040	0.020	Beta	[26, 27]
HCC-DTHLR [year 1]		0.427	0.049	Beta	[28]
HCC-DTHLR [year 2]		0.427	0.049	Beta	[28]
LT1-DTHLR		0.116	0.029	Beta	[29]
LT2-DTHLR		0.044	0.010	Beta	[29]
F4-SVR-DC		0.008	0.003	Beta	[30]
F4-SVR-HCC		0.005	0.002	Beta	[30]
**Costs** ^a^ **Adverse event costs**					
Anemia		$ 13,000	±30%	Gamma	[31, 32]
Rush & Pruritus		$ 1,033	±30%	Gamma	[31, 32]
Depression		$ 3,366	±30%	Gamma	[31, 32]
**Hepatitis C drug prices** [**whole treatment**]					
DCV/ASV		$ 38,710	±30%	Gamma	Base case price
PR		$ 11,034	±30%	Gamma	[31]
PIs/PR^b^		$ 30,336	±30%	Gamma	[33]
**Monitoring costs** [**per week**]					
Week 0-24-48		$ 107	±30%	Gamma	[31]
Week 2–4		$ 125	±30%	Gamma	[31]
Week 8-16-20-28-32-40-44		$ 18	±30%	Gamma	[31]
Week 12		$ 62	±30%	Gamma	[31]
**Disease related costs**					
SVR [F0-F3] year 1		$ 56	±30%	Gamma	[31, 34]
SVR [F0-F3] year 2		$ 19	±30%	Gamma	[31]
SVR F4 year 1		$ 56	±30%	Gamma	[31, 34]
SVR F4 year 2		$ 325	±30%	Gamma	[31, 34]
F0-F1-F2		$ 190	±30%	Gamma	[31, 34]
F3		$ 282	±30%	Gamma	[31, 34]
F4		$ 420	±30%	Gamma	[31, 34]
DC1		$ 3049	±30%	Gamma	[24, 31, 32]
DC2		$ 2,489	±30%	Gamma	[24, 31, 32]
HCC1		$ 1,408	±30%	Gamma	[32]
HCC 2+		$ 956	±30%	Gamma	[31, 32]
LT1		$ 60,259	±30%	Gamma	[35, 36]
LT 2+		$ 8,262	±30%	Gamma	[31, 35, 36]
**Local utility measure** ^c^	**N° patients**	**Mean**	**SE**	**Distribution**	**Reference**
F0-F3	2	0.686	±30%	Beta	[20]
F4	9	0.682	0.285	Beta	[20]
DC	2	0.536	±30%	Beta	[20]
HCC	4	0.952	0.096	Beta	[20]
LT1	1	0.572	±30%	Beta	[20]
LT2+	2	0.904	±30%	Beta	[20]
SVR F0-F1	5	1	0	Beta	[20]
SVR F2-F3	2	1	0	Beta	[20]
SVR—F4	1	0.798	±30%	Beta	[20]
**Literature utility measure**					
F0-F1		0.77	0.19	Beta	[37]
F2-F3		0.66	0.15	Beta	[37]
F4		0.55	0.11	Beta	[37]
DC		0.45	0.10	Beta	[37]
HCC		0.45	0.10	Beta	[37]
LT1		0.45	0.10	Beta	[37]
LT2+		0.67	0.14	Beta	[37]
SVR F0-F1		0.82	0.19	Beta	[37]
SVR F2-F3		0.72	0.16	Beta	[37]
SVR—F4		0.72	0.16	Beta	[37]
**SVR**					
DCV/ASV		0.945	0.023	Beta	[19]
PR		0.489	0.018	Beta	[19]
PI		0.736	0.052	Beta	[19]

SE: Standard Error, PR: Peginterferon plus Ribavirin, PIs: Protease Inhibitors, DCV/ASV: Daclatasvir plus Asunaprevir, QALY: Quality Adjusted Life Years, DC: Decompensated Cirrhosis, HCC: Hepatocellular carcinoma, LT: Liver transplant, SVR: Sustained viral response, DTHLR: Disease specific death, CENABAST: National public drug supplier

^a^All costs were adjusted to 2014 US dollars and rounded to the nearest hundred.

^b^ Price corresponds to the average 2014 price.

^c^EQ-5D survey to local patients was valued using Chilean published tariff.

#### Chilean patient profile

In 2004 a descriptive study conducted in Chile reported epidemiological and clinical characteristics of patients infected with HCV. The average age of diagnosis was 54 years and the main risk factor for infection was blood transfusion. From the 6 HCV genotypes identified, genotype 1b was the most prevalent (82%) followed by genotype 3a (12%) [17].

#### Health states transition probabilities

Transition probabilities were obtained from a cost effectiveness literature review in Hepatitis C. The inputs data were assessed considering generalizability and transferability aspects, which were validated by local clinical experts. Hence, they were considered a good estimate to best reflect the reality of most Chilean patients with Hepatitis C (Table 1).

#### Effectiveness of treatment strategies

Because no "head-to-head" studies comparing DCV/ASV versus PR and PIs/PR is available, data from a network meta-analysis (NMA) was used [19]. The main objective of this study was to compare the results of efficacy and tolerability of DCV/ASV, PR, PIs/PR among other alternatives, in different groups of patients. From the reported odds ratio, probability transitions were calculated representing the proportion of patients who achieved SVR after treatment (Table 1).

#### Quality of Life

Health benefits were measured in quality adjusted life years (QALY) using local data. The EuroQol five dimension generic questionnaire (EQ-5D) was administered to a total of 28 patients in different states of the MONARCH model. The study was approved by the ethics commitee of the faculty of Medicine at Pontificia Universidad Católica de Chile. All patients were recruited at the “Hospital Clínico Universidad Católica” and each patient signed the corresponding informed consent approved by the ethics committee of the Faculty of Medicine at Pontificia Universidad Católica de Chile. In addition, the interviewer completed a survey using the patient’s clinical records to assess the current state of the disease. The results of each of these surveys were valued using the Chilean tariff [20], which used the Time Trade Off (TTO) valuation methodology (Table 1). Utilities reported in the literature for all health states in the model were used to assess the impact in the ICER through a deterministic sensitivity analysis.

#### Costs

Costs were measured from the perspective of the public health system in Chilean pesos (CLP) (adjusted to 2014 using the consumer price index) and then converted to US Dollar (1 $US = 620 CLP). Different sources of information were used to identify the utilization of resources for each health state representing the natural history of the disease. The main source was the last version of Hepatitis C treatment Guidelines developed by the Ministry of Health of Chile in 2010 [34]. This data was supplemented with information reported in the English and Spanish guidelines [38, 39].

The valuation of resources was performed using the 2012 verification cost study performed by the Ministry of Health of Chile [40] and the 2014 FONASA tariff for public provision of health services (Modalidad de Atención Institucional, MAI) [32]. The price of treatments with no coverage in the health system was obtained from the national public drug supplier (CENABAST), which provided average sales prices during 2014 (Table 1). The study followed the Chilean guidelines for economic evaluation provided by the Ministry of Health [41].

Because there is no price set by the manufacturer to date, the cost-effectiveness analysis was performed considering four possible total drug prices: $US 77,419, $US 58,065, $US 38,710, and $US 19,355 (i.e. per week cost between $US 806 and $US 3,226). The highest price corresponds to the most expensive one observed in the international market. The remaining prices correspond to the 75%, 50% and 25% respectively.

## Results

An incremental analysis was performed to determine the cost effectiveness of DCV/ASV versus PIs and PR. The same efficacy was assumed between the two possible PIs (boceprevir and telaprevir), and the price was assumed to be the lowest observed in the local market. The results showed that when assuming a DCV/ASV total treatment price of $US 77,419 (the highest price analysed), PIs are a possible efficient comparator with an ICER of $US 11,977 versus $US 19,790 per additional QALY for DCV/ASV. However, it is unlikely that the Chilean Health system will pay the highest price given the wide range of prices of new drugs for hepatitis C observed international markets. Hence, we followed the analysis with the second highest price ($US 58,065), in which case PIs were extendedly dominated by DCV/ASV and PR (see Table 2). Extended dominance means that the additional QALY gained is more expensive with PIs than DCV/ASV [42]. Because this has been observed with the second highest price examined, any lower price of DCV/ASV will locate PIs above the efficiency frontier. Hence, the relevant ICER to inform decision making about allocative efficiency was estimated by comparing DCV/ASV against PR.

**Table 2:** Incremental cost effectiveness analysis: DCV/ASV versus PR and PIs considering two prices.

Treatment strategy	Total drugCost ($US)	Total intervention cost ($US)	Total QALY	Incremental cost ($US)	Incremental QALY	ICER ($US/QALY)
PR	11,040	17,803	10.9	-	-	-
PIs	30,335	34,599	12.3	16,796	1,40	11,977
DCV/ASV	77,419	80,117	14.6	45,518	2,3	19,790
DCV/ASV	58,065	60,775	14.6	26,176	2,3	11,380
DCV/ASV	38,710	41,856	14.6	7,257	2,3	3,155

PR: Peginterferon plus Ribavirin, PIs: Protease Inhibitors, DCV/ASV: Daclatasvir plus Asunaprevir, QALY: Quality Adjusted Life Years, ICER: Incremental cost effectiveness ratio, 1 $US = 620 CLP.

### Base case scenario

The cost-effectiveness results of DCV/ASV versus PR are shown in Table 3 considering four possible prices of the new drug. However, a base case scenario was defined at a total treatment price of $US 38,710 per patient. It was estimated that one patient treated with DCV/ASV was expected to gain 2.2 additional life years and 3.7 additional QALYs versus patients treated with PR over the course of his/her life. On the other hand, the total expected cost of treating one patient diagnosed with Hepatitis C genotype 1b was $US 41,856 for DCV/ASV and $US 17,803 for PR (incremental cost of $US 24,053). This difference was mainly explained by the price of the new drug. In the case of DCV/ASV, 90.7% of the total cost of the intervention is due to the drug. In contrast, if the cohort is treated with PR only a 51% of the total intervention cost is due to the drug while 44.3% corresponded to the expenditure for the management of the disease and its complications. The estimated ICER was $US 6,375 per additional QALY, which can be considered cost-effective for a reference threshold value of 1 Gross Domestic Product (GDP) per capita suggested in the Chilean economic evaluation guideline [41].

**Table 3:** Probabilistic sensitivity analysis considering four DCV/ASV prices.

	Total treatment cost ($US)	Total cost DCV/ASV ($US)	Total QALY DCV/ASV	Total cost PR ($US)	Total QALY PR	ICER^a^ ($US/QALY)	Prob. of cost-effectiveness^b^
**Local utility estimates**	77,419	80,117				16,635	43,6%
	58,065	60,775	14.6	17,803	10.9	11,581	71,5%
	38,710	41,856				6,375	91,6%
	19,355	22,920				1,364	98,6%
**Literature utility estimates**	38,710	41,958	12.6	17,921	10.08	9,241	86.3%

PR: Peginterferon plus Ribavirin, PIs: Protease Inhibitors, DCV/ASV: Daclatasvir plus Asunaprevir, QALY: Quality Adjusted Life Years, ICER: Incremental cost effectiveness ratio, 1 $US = 620 CLP

^a^Montecarlo simulation (5,000 iterations)

^b^Considering a suggested threshold of 1times GDP per capita

The ICER ranged from an average maximum of $US 16,635 per additional QALY (at a total treatment price set at $US 77,419) to an average minimum of $US 1,365 per additional QALY (at a total treatment price set at $US 19,355).

The economic model estimated the occurrence of hepatitis C complications in naive patients for all drug combinations. It was estimated that 17.8% (BCI 95% 17–18.5%) of the patients developed liver cirrhosis when treated with PR versus 1.9% (BCI 95% 1.2–2.8%) when treated with DCV/ASV alongside the course of their lives. In terms of major liver complications, DC occurred in 26.6% (BCI 95% 23.1–31.7%) of the patients treated with PR and 2.9% (BCI 95% 1.7–4.6%) with DCV/ASV. Furthermore, it was estimated that 14.4% (BCI 95% 7.7–18.5%) of patients developed HCC when treated with PR versus 1.5% (BCI 95% 0.7–2.4%) when treated with DCV/ASV. Similarly, a difference was observed when estimating the cumulative incidence of LT (3.2% and 0.3% in patients treated with PR and DCV/ASV respectively) (see Table 4).

**Table 4:** Expected Hepatitis C complications in patients treated with DCV/ASV versus PR.

	DCV/ASV				PR			
	F4 (%)	DC (%)	HCC (%)	LT (%)	F4 (%)	DC (%)	HCC (%)	LT (%)
**% patients developing HCV complications**	1.90	2.85	1.54	0.34	17.75	26.59	14.39	3.20
**BCI 95%**	1.2–2.8	1.7–4.6	0.7–2.4	0.1–0.6	17–18.5	23.1–31.7	7.7–18.5	1.6–4.6

HCV: Hepatitis C Virus, PR: Peginterferon plus Ribavirin, PIs: Protease Inhibitors, DCV/ASV: Daclatasvir plus Asunaprevir, F4: cirrhosis, DC: Decompensated cirrhosis, HCC: hepatocellular carcinoma, HT: liver transplant, BCI: Bayesian credibility interval.

### Sensitivity analysis

**Fig 2:**
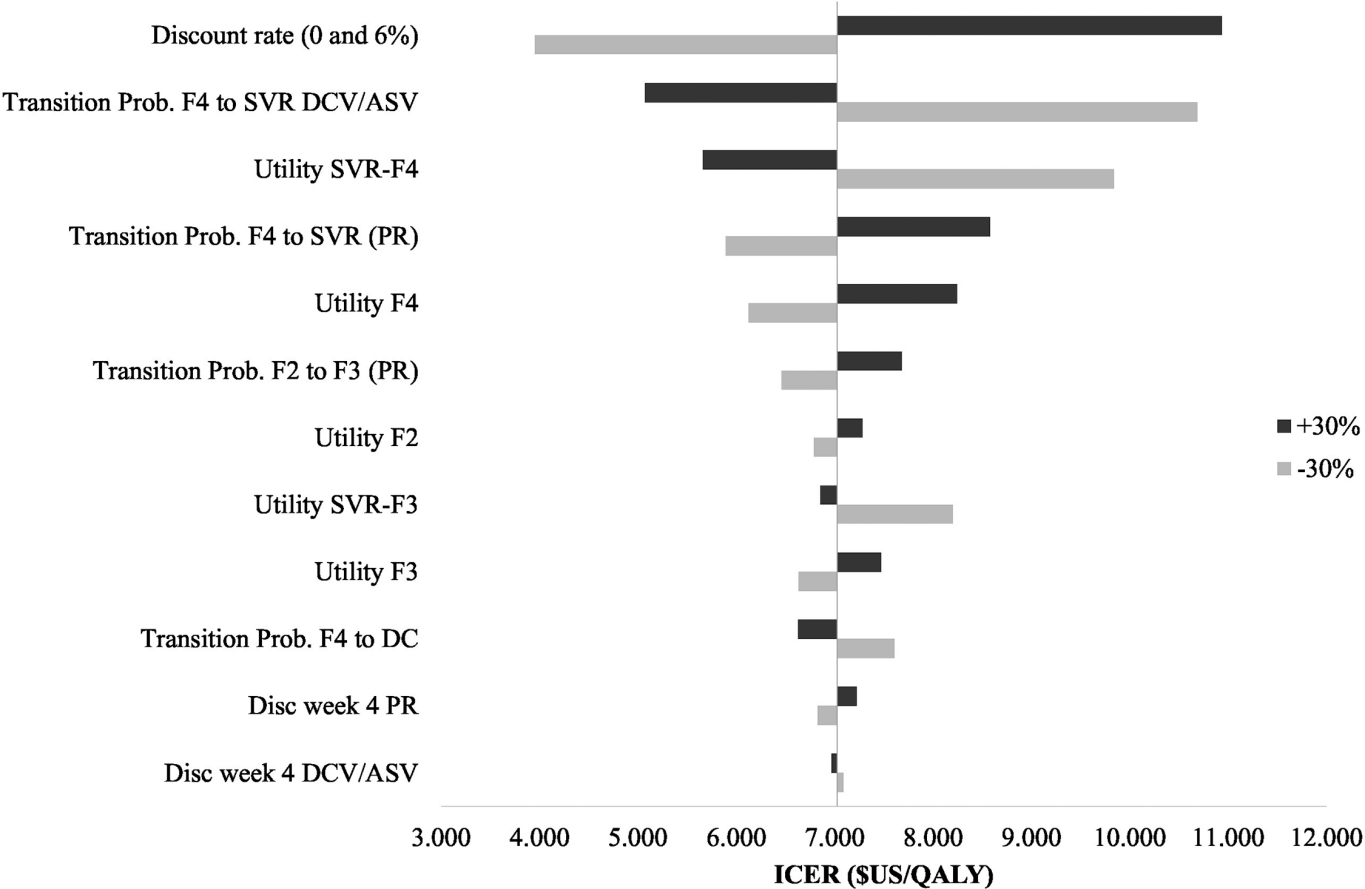
Deterministic sensitivity analysis tornado graph: Tornado graph showing the variation of the ICER when parameters are varied independently. The parameters with highest impact on the ICER are shown on the graph: transition probability from F4 to SVR when the treatment is DCV/ASV and PR, transition probability from F2-F3, transition probability from f4 to DC, QALY of the SVR-F4 and SVR-F3 states, QALY of the F2, F3 and F4 state, treatment discontinuation at week 4 for PR and DCV/ASV.

A one-way sensitivity analysis was performed for transition probabilities, QALYs, discount rates, disease related costs, treatment costs and other variables considering the base case scenario as a benchmark. The objective of this analysis was to determine the variation of the ICER of the base case scenario when parameters vary independently. The results of these variations are presented on the tornado graph (Fig 2). The biggest impact on the ICER occurred when the discount rate was modified to 6% and 0%, reaching values of US$ 10,933 and US$ 3,952 per additional QALY respectively. The rest of the parameters were modified by ± 30% in relative terms. A 30% increase of the transition probability from F4 to the state of SVR when treated with DCV/ASV (equivalent to a SVR rate of 100%), reduced the ICER to US$ 5,662 per additional QALY, whereas a reduction of 30% produced and increase of the ICER up to US$ 9,839 per additional QALY. Likewise, the same variation in the transition probability for PR changed the ICER from US$ 5,893 to US$ 8,579 per QALY gained.

**Fig 3:**
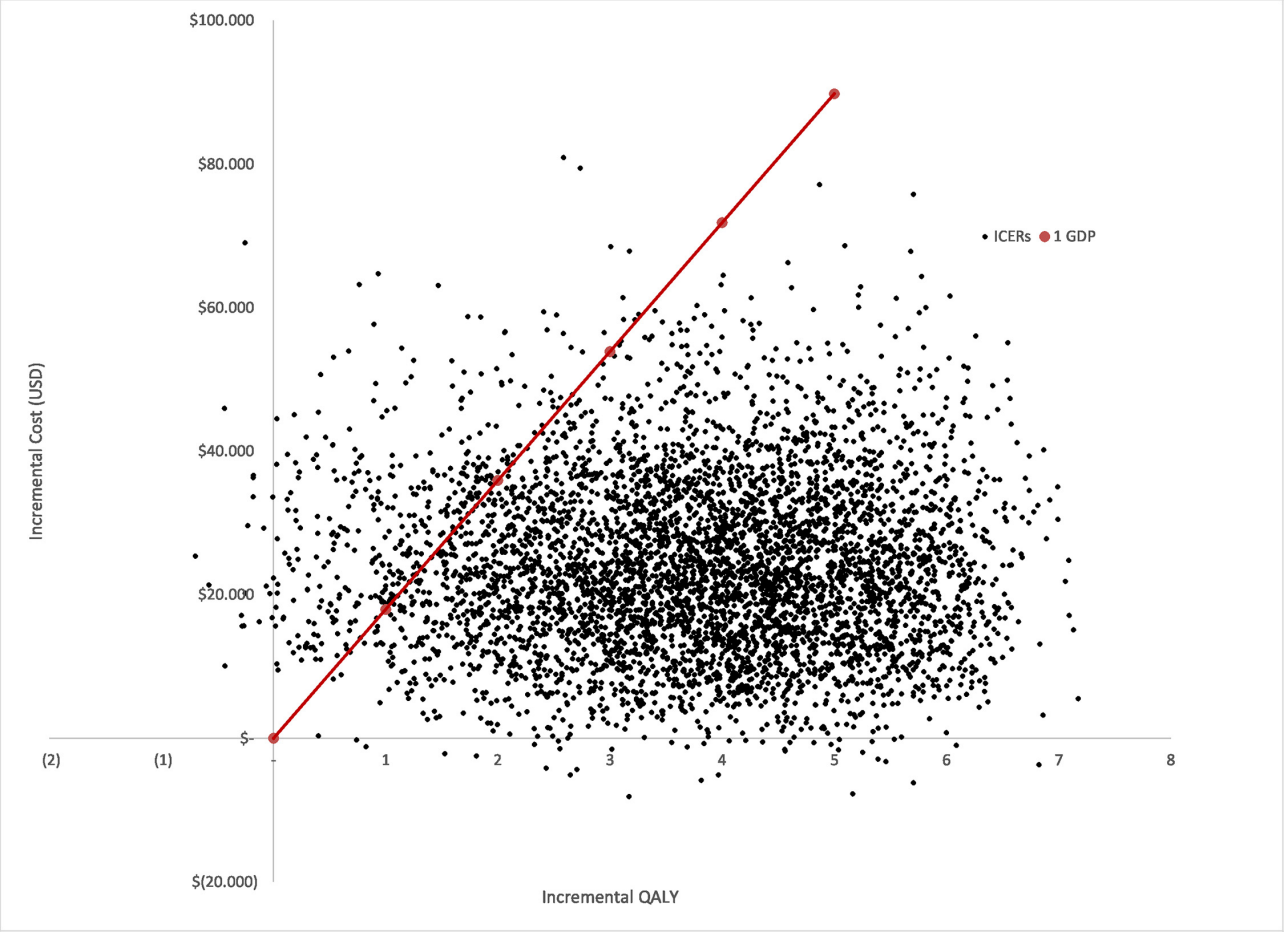
Cost-effectiveness plane for DCV/ASV versus PR: The cost-effectiveness plane represents the base case scenario assuming a DCV/ASV total treatment price of US$ 38,710.

**Fig 4:**
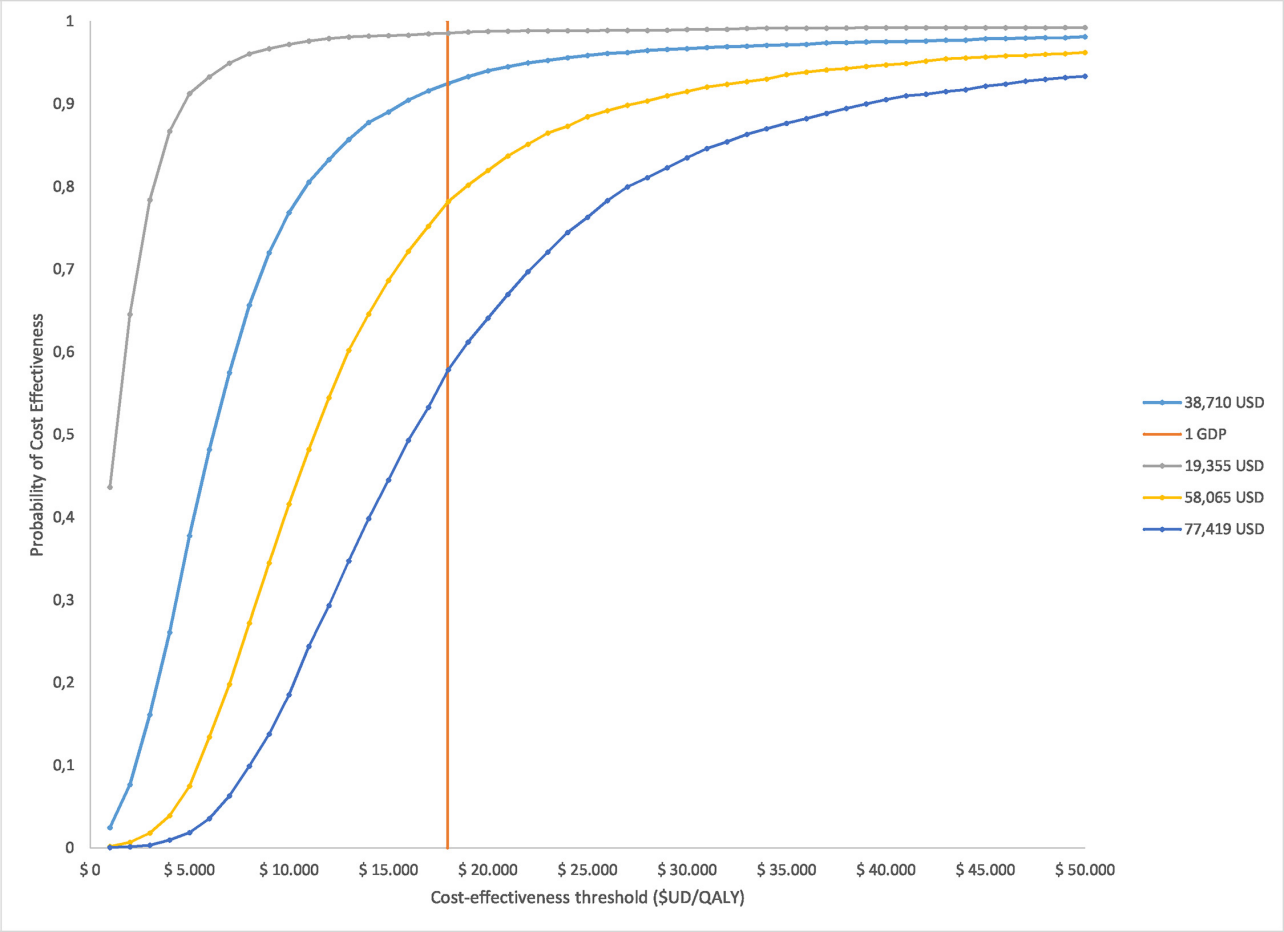
Acceptability curves for DCV/ASV versus PR for different prices of DCV/ASV: The acceptability curve shows the probability of cost-effectiveness considering four different prices for DCV/ASV (total drug treatment cost): US$ 77,419—US$ 58,065- US$ 38,710- US$ 19,355.

A probabilistic sensitivity analysis (PSA) was performed to characterize the second order uncertainty of all parameters included in the model (Table 3). 5000 Monte Carlo simulations were carried out. Fig 3 shows that most iterations lie on the north-east quadrant of the cost effectiveness plane. The graph also shows the proportion of the iterations for which the intervention is cost effective considering different threshold values (1 and 2 GDP per capita). Cost effectiveness acceptability curves are presented for four different prices in Fig 4. Whilst the probability that DCV/ASV is cost effective is 91.6% at a price of US$ 38,710, there is a 21.3% probability that the intervention dominates PR (lower total cost and more effective) when the price s US$ 19,355.

Because the utility values used to estimate QALYs were based on a small sample of patients, we conducted a secondary analysis with values obtained from a literature review [37] (Table 3). Considering this new set of utilities, the ICER increased from US$ 6,375 to US$ 9,241 per additional QALY (31%), which is still considered cost-effective, and reduced the probability of cost effectiveness from 91.6% to an 86.3%.

## Discussion

Until 2011 the only treatment available for Hepatitis C patients was PR. This treatment is associated with low rates of SVR and a poor safety profile impacting negatively the quality of life of patients. However, a group of new molecules have been developed in the last few years. They have changed the prognosis of the disease in terms of efficacy, safety and quality of life, raising the question whether health systems should pay for these new interventions. This research provides valuable information to health decision makers to address this question.

The use of DCV/ASV substantially reduced the disease burden of Hepatitis C. It showed a significant effect on decreasing the number of complications such as DC, HCC and LT, with a reduction greater than 89% in all cases. These results led to important savings on future costs associated with disease complications that occur because of the reduced effectiveness of PR relative to DCV/ASV. Consequently, 44.3% of the total intervention costs with PR were associated with the provision of health care in patients who progressed to more advanced stages of the disease, which contrast with the 8.3% in patients treated with DCV/ASV. Furthermore, health benefits from the new strategy arose mainly from the increased SVR, improved safety profile, enhanced quality of life and shorter treatment duration.

The results of this study showed that DCV/ASV is cost-effective for the Chilean public health system even if the highest price assessed in this study was paid. Moreover, the deterministic sensitivity analyses showed that variations of most parameters impacted marginally the cost-effectiveness. In particular, the variation of the discount rate between 0% and 6%, which had the largest impact on the ICER, did not affect the decision of considering DCV/ASV cost-effective in the base case scenario (total treatment price of $US 38,710). In addition, when key clinical and cost parameters (such as SVR rates, adverse event incidence, treatment and monitoring costs, discontinuation rates and transition probabilities) were adjusted across plausible ranges, the strategy continued to be cost-effective showing robustness of the results. On the other hand, the stochastic analysis showed that the probability of cost effectiveness was more than 90% at a total treatment price of 38,710 and nearly a 100% at a price of $US 19,355. More importantly, at this latter price (the cheapest price assessed) there is a probability of 21.3% that DCV/ASC dominates PR (i.e. on the long run the public health system saves money if it pays for the new treatment).

The results of this study are consistent with other recently published cost-effectiveness studies evaluating new generation of oral HCV treatments. Nevertheless, to our knowledge, this is the first study examining the cost-effectiveness of DCV/ASV versus PR and PIs. A recent study was conducted in the United States compared triple therapy with PIs with sofosbuvir/PR and three other PR free regimens: sofosbuvir/simeprevir, sofosbuvir/daclatasvir, and sofosbuvir/ledipasvir from a societal perspective [43]. This study revealed that novel hepatitis C treatments were cost-effective compared with usual care in genotype 1 but not other genotypes. Similarly, in a US setting, treatment with sofosbuvir/ledipasvir versus standard of care (PR and PIs/PR) was also found cost-effective only in genotype 1 patients at a threshold of $US 50,000 per additional QALY [44]. Furthermore, another study was conducted to determine the cost effectiveness of treating patients at early stages of the disease versus delaying treatment until progression to the next disease stage occurs. This study, which assumed equivalent efficacy among all novel hepatitis C drugs (above 90%), showed that immediate treatment with these new drugs was cost-effective in patients with moderate to severe liver disease (F2-F4) [45].

An important strength of this study is that quality of life estimates were obtained from Chilean patients through the EQ-5D survey and valued according to the published Chilean tariff [20], though it faces the limitation of a small sample size. We included these estimates in the base case scenario because the Chilean health system values the fact that QALYs were estimated from local data even though the total number of participants can be considered insufficient from standards of classical statistical inference. In order to account for this uncertainty we explored the impact of variations of these estimates in the deterministic sensitivity analysis, and the overall second order uncertainty in the probabilistic sensitivity analysis. The results indicated that the decision would not be affected because of these parameters. In addition, we conducted a complementary analysis using utility values from the literature. It was found that in Chile, patients with HCV infection provided higher values for the SVR health state compared to international published literature [37, 46, 47]. We observed a 31% increase of the ICER (from US$ 6,375 to US$ 9,241 per additional QALY) in the base case, when using utility values from the literature versus those locally measured. This result reduces the probability of cost effectiveness from 91.6% to 86.3% but does not affect the decision. In other words, the use of values obtained from the literature may underestimate the impact on health related quality of life (HRQoL) and cost-effectiveness of the new treatment. This element impacted positively on the cost-effectiveness of DCV/ASV and suggests that local information should be always considered given the important differences between the ICERs.

On the other hand, it must be acknowledged that the model cannot capture all the elements related to the epidemiological dynamics of the disease. For example, the treatment of all patients with HCV infection today with a very effective treatment, will affect the rate of infection in the future. This is expected to decrease the incidence and eventually eradicate the illness. This type of positive externality is not captured in the model and it might determine significant future saving. The current model implicitly assumes that the prevalence and incidence will not vary in the future, which underestimates the overall positive effects of the new technology.

As any economic study relying on aggregate data, our study faces the limitation of combining different sources of information, each entailing different degrees of uncertainty. However, all this second order uncertainty was characterized and propagated through a probabilistic sensitivity analysis, showing high probability of cost-effectiveness. On the other hand, it is important to acknowledge the limitation of characterizing heterogeneity, i.e. how disease progression or cost-effectiveness itself can be determined by different factors such as gender, race, alcohol consumption and various co-morbidities. Future research might focus on revealing the value of this new treatment for particular subgroups [9].

All the cost effectiveness evidence presented in this analysis responds specifically to genotype 1b patients. This was considered a reasonable approach considering the characteristics of hepatitis C in Chile, were more than 80% of patients corresponds to this category [17]. In addition, the efficacy of DCV/ASV reported in clinical trials is mainly on this particular subgroup of patients [14, 15]. However, cost effectiveness evidence of other novel hepatitis C with improved efficacy on other genotypes (mainly 3 and 1a), would also be valuable to inform decisions since they are also at risk of disease complications.

In terms of generalizability of the results, the current study has some limitations. First, the unit costs are local and might differ significantly from other jurisdictions, though they might be similar to other upper-middle or low-high income countries. In addition, the perspective adopted in this study followed the Chilean guidelines for economic evaluation and did not include indirect costs. Second, as explained before, our study collected local data to estimate QALYs. This strength for its use in Chile may be considered a limitation in terms of generalizability, although it depends on local judgments. For example, there is evidence that Chilean values based on EQ-5D are similar to the values reported on British and Spanish populations, whereas they differ from those reported from Latin-American residents in the US or Argentinians [20]. Third, experts validated the model used in this study, which was considered a good representation of the health problem, in particular to examine progression from early stages of the disease such as in Hepatitis C. Hence, in terms of the structure of the model, we consider our study is highly generalizable. Finally, local experts also validated the transition probabilities used in the model, which can be also valid for other countries. Therefore, the generalizability of these data should be judged according to the epidemiology of the jurisdiction where this study expects to be used as a source of information.

## Conclusion

In conclusion, the combination of DCV and ASV can be considered cost-effective for the Chilean health care system compared to PIs and PR. These results provide relevant information about the value of incorporating these drugs into the public Chilean healthcare system and may be useful to inform coverage decisions on other middle income countries.
